# Non-Cognitive Predictors: Evidence and Implications for Academic Achievement and Cognitive Processing

**DOI:** 10.3390/jintelligence13100133

**Published:** 2025-10-21

**Authors:** Lazar Stankov, Jihyun Lee

**Affiliations:** 1 School of Psychology, The University of Sydney, Camperdown, NSW 2050, Australia; 2 School of Education, The University of NSW, Sydney, NSW 2033, Australia; jihyun.lee@unsw.edu.au

**Keywords:** social/emotional skills, big five personality, socio-economic status (SES), self-beliefs, self-efficacy, test anxiety, confidence

## Abstract

This is a review of recent findings about the role of non-cognitive variables in predicting academic achievement. Many indices considered up until now, including the Big Five personality measures, have low predictability. This has been supported by the findings from previous TIMSS and PISA large-scale surveys and recent studies based on measures of Social and Emotional Skills. Socio-economic status remains a good predictor but also crucial are psychological measures of self-beliefs (self-efficacy, test anxiety, and confidence).

## 1. Introduction

Psychological processes of interest to students of individual differences tend to have an applied aspect. For example, research on intelligence and personality is seen as being important for clinical and many work-related activities. From the very beginning of contemporary studies of intelligence, education has also been an important area of application. Economists and policymakers have become interested in using psychological measures to predict what they refer to as life outcomes (e.g., income, well-being, health, and educational success). Recently, two teams of economists became involved in an argument about the relative importance of personality and cognitive ability for the prediction of these criteria (see Stankov 2023). Borghans et al. (2016) claimed that non-cognitive measures, including Big Five personality scales, are good predictors of grades and school achievement. This was rebutted by Zisman and Ganzach (2022) who published a paper in the *Intelligence* journal entitled, “The claim that personality is more important than intelligence in predicting important life outcomes has been greatly exaggerated”.

There can be no doubt that both are important (Tetzner and Rammstedt 2020). For example, a recent paper by Demetriou et al. (2023) produced evidence for the changing roles of personality and intelligence in school performance during the early years of life (i.e., from preschool to adolescence). On the other hand, Sanchez-Izquierdo et al. (2023) showed that intelligence acts as a protective life outcome factor prior to reaching upper-middle age, but survival in old age depends more on non-cognitive processes operating during the lifespan.

A critical question from a psychologist’s point of view is which specific aspects of personality and intelligence predict academic achievement and other life outcomes. The focus of this paper is on non-cognitive measures that encompass both personality and contextual variables, like those captured by socio-economic status (SES). The question it addresses is which non-cognitive variables predict an important life outcome of academic achievement. This paper is a narrative review; it does not apply any systematic methodology. A systematic recent review of PISA data by Wang et al. (2023) covers both the psychological processes of individual students and broader influences on achievement such as household context, school community, education systems, and macro society. Their analyses uncovered seven factors that are consistently associated with math achievement. Two of these—student grade level and overall family SES—are positively associated with math achievement. The remaining five factors are consistently negatively associated with math achievement: student absenteeism and lack of punctuality, school repeating and dropout rate, school prevalence of students’ misbehavior, shortage of teachers, and student-centered instruction.

The present review will consider empirical findings from recent studies carried out by our team using data available from both large-scale studies and our own empirical research. This work was not experimental in nature. It was based on data collected in surveys containing questionnaires that measure a range of non-cognitive constructs. Descriptive statistics for most statistical analyses were based on measures of correlation which were followed by multivariate procedures, including regression and factor analysis, as well as versions of structural equation modeling. This review focuses on the relationships between a range of non-cognitive factors and both academic achievement and cognitive processes. The outcome variables under consideration are cognitive in nature.

The first section will summarize findings with non-cognitive constructs and measures that tend to have small correlations (i.e., effect size) with achievement scores, since most of the studies to be considered have large samples of participants and therefore even small correlation coefficients (r < 0.05) may be declared statistically significant. The second section will point to the constructs that have a noteworthy relationship with achievement scores. In the remaining parts of the paper, some practical and theoretical implications of our findings to date will be mentioned.

## 2. Ignorable Non-Cognitive Predictors of Academic Achievement

This section considers findings with the variables that are seen as important for education since they are believed to be affecting the process of learning, but, in fact, empirical findings indicate that they are not effective for this purpose. One set of findings to be considered is based on a broad range of indices gathered by the international studies of educational achievement. The second set of findings is narrower since it covers the results from studies that have employed only one instrument—the now-popular Big Five measure of personality.

Instead of using Cohen’s (1988) suggestions for criteria to be utilized in the evaluation of effect sizes, we relied on the more recent proposal of Funder and Ozer (2019) who suggested that correlations of .05 and .10 are small, although the latter may be consequential under some circumstances. They state that “…an effect-size r of .20 indicates a medium effect that is of some explanatory and practical use …, and an effect-size of r = .30 indicates a large effect that is potentially powerful in both the short and the long run. A very large effect size is r = .40 or greater…” (p. 156). However, we contend that correlations up to .20 should be considered small (despite containing a section of ‘medium’ in Funder and Ozer 2019), partly because variables having below .20 correlations tend to have insignificant loadings in factor analyses due to large unique rather than common variance (Stankov 2013). For correlations higher than .20, Funder and Ozer’s (2019) limits align reasonably well with our interpretations in the reviewed studies: *medium* effect sizes for correlations between .20 and .30, whereas *large* were between .30 and .45 and *very large* above .45.

### 2.1. Variables Employed in PISA and TIMSS Surveys

Large-scale studies, like the Programme for International Student Assessment (PISA) and the Trends in International Mathematics and Science Study (TIMSS), carried out by international bodies contain information about performance on tests of various school subjects, especially mathematics, obtained from large samples of participants (i.e., more than half a million students and more than 70 countries in recent PISA surveys). Extensive information about participants, parents, schools, and teaching practices is also collected. This includes specific questions (some are listed in parentheses below) from PISA (45 variables) and TIMSS (24 variables) datasets that can be classified into 13 domains of educational psychology. Lee and Stankov (2018) reported correlations between all 69 variables and mathematics achievement scores, and it was remarkable that 56 of them were small, i.e., lower than r = .20. Only two domains—*Self-beliefs* and *School Climate*—contained variables with medium and above effect sizes. Non-cognitive variables that were classified into the remaining eleven broad domains with small effect sizes are as follows:

- *Personality traits* (Openness to problem solving, Perseverance, Mathematics work ethic, and Sense of belonging to school).
- *Curriculum exposure* (Math specific activity, Traditional classroom activity in math, Traditional classroom activity in science, Science experiment activity, Familiarity with mathematical concepts).
- *Affect* (Positive affect towards math).
- *Vocational interests* (Labor market information, Information about careers).
- *Motivational factors* (Student engagement in math lesson, Instrumental motivation for mathematics, Mathematics interest, External attributions to failure in mathematics).
- *Planned behavior* (Attitude towards school in terms of learning outcomes and learning activities, Mathematics behavior, Mathematics intentions, Subjective norms in mathematics).
- *Learning/instructional time* (Time spent on extra math lessons, Total minutes of instructional time per week, Learning time in mathematics, Out-of-school study time, Ratio of math and total instructional time).
- *Value* (Valuing math).
- *Learning strategies* (Competitive learning, Co-operative learning, Use of control strategies, Use of elaboration strategies, Use of memorization strategies).
- *Teacher Behavior* (Math teacher’s classroom management, Cognitive activation in mathematics lessons, Teacher–student relations, Mathematics teacher’s support, Teacher-directedness, Teacher behavior: Formative assessment, Teacher behavior: Student orientation).
- *Homework* (Weekly time spent on math homework, Weekly time spent on science homework, Relative time spent on math homework).

Put simply, none of the above eleven domains or answers to the actual questions listed within the parentheses predict mathematics achievement at a noteworthy level. This is even though many, or indeed all, questions were included in the surveys because there was a premeditated belief that they are important for learning academic subjects like mathematics.

Recent studies based on large-scale datasets have been focused on specific countries and/or academic areas. For example, using PISA 2018 data, Karaman (2022) has shown that meta-cognitive strategies were the most influential non-cognitive variables for predicting reading achievement in Turkey. Michaelides et al. (2019) focus on motivational profiles in TIMSS mathematics.

#### 2.1.1. Big Five Personality Traits

Over the past couple of decades, a popular way to assess ‘normal’ personality structure (see McCrae et al. 2005) has been based on the Big Five personality test consisting of the following main scales: Openness, Conscientiousness, Extraversion, Agreeableness, and Neuroticism (or Emotional Stability). This test was deliberately constructed to avoid any overlap with the measures of intelligence and indeed Stankov (2018) has shown that all Big Five scales have low correlations with the Number Series test of fluid intelligence. Since these scales also have low correlations with measures of the decision-making processes, social conservatism, and self-beliefs, it was suggested that the domain of personality should be expanded to include new trait measures.

Only Openness to experience occasionally correlated higher than r = 0.20 with cognitive tests and on one occasion it reached a correlation close to r = 0.30 with academic grades (see Stankov 2018). In the studies of others, Conscientiousness was also found to correlate around r = 0.20 with achievement (see Poropat 2009).

This was supported by the meta-analyses of Meyer et al. (2023) and Mammadov et al. (2021) presented in their Table 1. Inspection of the values within that table shows again that Big Five measures of Conscientiousness (r = .20) and Openness (r = .13) have the highest but nevertheless comparatively low correlations with academic achievement. The other three Big Five measures correlate close to zero with it (Extraversion r = .01, Agreeableness r = .06, and Neuroticism r = −.02). Included in the above three meta-analyses were measures of cognitive ability/intelligence which had the average correlation of r = .34 with academic achievement. Roth et al. (2015) point out that intelligence is the strongest predictor of school achievement, and the correlations range from r = .30 to r = .70, with mean correlation being r = .54. The variability of these correlations is dependent in part on the cognitive measures employed—fluid intelligence tasks may correlate differently with academic achievements than crystalized intelligence tasks. For the purposes of the present paper, we can note that all Big Five non-cognitive measures have significantly lower correlations with achievement than measures of cognitive ability/intelligence. Poropat’s (2009) meta-analysis agreed that Conscientiousness correlates close to .20 (i.e., r = .22) with academic achievement but he reported an unusually low (r = .25) and unexplained correlation between intelligence and academic achievement.

It is also useful to note that correlations between personality traits and academic achievement vary depending on the measure of achievement employed. For example, Meyer et al. (2023) report that different correlations were obtained for the domains of language (e.g., English, German, etc.) and STEM (Science, Technology, Engineering, and Mathematics). They also report that Openness has a raw correlation r = .20 with language scores and r = .10 with STEM scores. Conscientiousness, however, has almost identical correlations (r = .19 and r = .18) with language and STEM scores. Also, different correlations may be obtained with school marks and standardized knowledge test scores. Thus, Conscientiousness correlates r = .23 with school grades and r = .11 with STEM test scores. Openness, however, correlates equally (r = .17) with school grades and STEM test scores.

There is now additional evidence that Big Five scales are not particularly useful for the prediction of important life outcomes. Anglim et al.’s (2022) meta-analysis found that Openness (ρ = .20) and Neuroticism (ρ = −.09) were the strongest Big Five correlates of intelligence and that Openness correlated more with crystallized than it did with fluid intelligence.

Stankov (2023) reports correlations between the Big Five scales and measures of Educational Achievement and income (log pay) collected during midlife. The analysis was based on Zisman and Ganzach’s (2022, see their Appendix A.7) report. The highest correlation is r = .15 between Educational Achievement and Openness. The remaining correlations are lower than r = .10. Again, personality traits measured by the Big Five scales are poor predictors of a midlife outcome.

#### 2.1.2. Social and Emotional Skills Assessment

Major testing establishments like the Organization for Economic Co-operation and Development (OECD) and the Educational Testing Service (ETS) are advocating the use of the Big Five domains as a framework for developing scales to be used in large surveys of Social and Emotional Skills (see Abrahams et al. 2019). An OECD report by Kankaraš and Suarez-Alvarez (2019) has re-named the Big Five domains (e.g., Conscientiousness became Social and Emotional Skill of Task Performance). A recently published OECD (2024) study reported findings with 15-year-old high school students from 16 countries and the results do not appear encouraging.

Altogether, 15 scales of Social and Emotional Skills were constructed. They are listed below following the Big Five domains they are supposed to capture.

- Conscientiousness (or Social and Emotional Skill of Task Performance) scales: Responsibility (b = 0.120), Self-control (b = 0.106), Persistence (b = 0.153), Achievement motivation (b = 0.201).
- Emotional Stability (or Social and Emotional Skill of Emotional Regulation) scales: Stress resistance (b = 0.010), Optimism (0.049), Emotional control (b = 0.058).
- Extraversion (or Social and Emotional Skill of Engaging with Others) scales: Sociability (b = −0.021), Assertiveness (b = 0.070), Energy (b = 0.037).
- Agreeableness (or Social and Emotional Skill of Collaboration) scales: Empathy (0.067), Trust (b = 0.033).
- Openness (or Social and Emotional Skill of Open-mindedness) scales: Curiosity (b = 0.144), Tolerance (b = 0.074), Creativity (b = 0.058).

The OECD (2024, see their Table 4.1) publication presents the results of multiple regression analyses that employed achievement scores in Mathematics, Reading, and Arts as criteria and all 15 skill scales listed above as predictors. The average standardized regression coefficients (b) for each skill are provided within the parentheses above. When there is only one predictor and one criterion, b is the same as the correlation coefficient between these two variables. With multiple predictors, b indicates a percentage increase in criterion if the predictor itself changes one standard deviation. For the purposes of the present paper, we use b = 0.200 as a threshold for noteworthy relationships. As an illustration, consider two IQ tests whose standard deviations are 15 IQ points. In this case, b = 0.200 implies that a 15 IQ point-change in one test leads to a substantively small 3 IQ point-change in the other test (i.e., 15 × 0.200). An important feature of the standardized regression coefficients in multiple regression is that they can be compared with the other coefficients.

As can be seen above, 14 Social and Emotional Skill scales have regression coefficients lower than b = 0.200, implying that all of them have low relationships with academic achievement. This is a disappointing finding for those advocating the use of Social and Emotional Skill scales for prediction purposes. Only one Social and Emotional Skill scale has a borderline noteworthy (b = 0.201) relationship with academic achievement in the OECD (2024) study. High achievement motivation refers to a person who sets high standards for oneself and works hard to meet them. Behaviorally, the person enjoys reaching a high level of mastery in some activity. It is worth noting that this skill belongs to the Conscientiousness domain which has also shown an occasional tendency to correlate with achievement scores. Conceptually, Achievement motivation may be related to the *Self-beliefs* domain (see below).

## 3. Noteworthy Non-Cognitive Predictors

In Lee and Stankov’s (2018) study, two educational psychology domains had larger than r = .20 raw correlations with mathematics achievement scores. They are as follows:

- *School climate* (Feeling of school safety, Disciplinary climate). Correlation of this domain with achievement in mathematics was r = 0.21, just above the adopted criterion value. It shows that students’ perception of school climate is related to successful academic performance.
- *Self-beliefs/Self-assessments* (Confidence with math, Mathematics self-efficacy, Mathematics self-concept, Mathematics anxiety, Educational aspiration, Expected educational level of student).

In addition, one non-cognitive domain that is usually listed as belonging to sociology or economy rather than educational psychology was identified as being important in Lee and Stankov’s (2018) study:

- *Socio-economic status* (SES): (Home possessions, Parental education, Highest educational level of parents, etc.).

Variables from the last two domains are important. They both have noteworthy correlations with achievement scores and some of the variables from the Self-beliefs domain have the highest correlations within the non-cognitive area. It is useful to consider the SES and Self-beliefs domains in more detail.

### 3.1. Socio-Economic Status (SES)

Lee et al. (2019) examined the relationship between family SES and academic achievement in PISA data. They concluded that the ten SES indices employed in PISA studies had correlations in the r = 0.20 s to 0.40 s range (p. 321). This is within the expectations suggested by previous work. For example, Sirin’s (2005) meta-analysis of the relationship between SES and academic achievement found a mean effect size of r  =  0.29. However, a more recent study by Harwell et al. (2016) reported a surprisingly modest result, with an average SES–achievement correlation of only r = 0.22. Lee et al. (2019) reported that the best predictive power for students’ mathematics achievement in PISA data was a composite measure of the economic, social, and cultural status (ESCS, r = 0.40), which was closely followed by another composite measure of a family’s educational and cultural home possessions (HOMEPOS, r = 0.36) as a proxy for family income. It can be concluded that SES has a noteworthy correlation with achievement scores and the availability of educational and cultural possessions within the family home may contribute to an improvement in academic achievement.

While the above-mentioned correlations and meta-analytic findings are based on raw correlations, Steinmayr and Kessels (2024) examined the incremental validity of SES for achievement after controlling for intelligence based on German primary and secondary school students. They assessed students’ intelligence, math and reading competencies, teacher grades, and family SES. The only domain where intelligence played a moderating role was in language arts teacher grades, while there was no moderating role of intelligence in the relationship between SES and mathematics or reading achievement. The authors concluded that “[the SES–achievement relationship] is mostly irrespective of students’ intelligence level. To put it differently, students academically benefit from a home with advantageous SES at all levels of intelligence” (p. 14).

### 3.2. Self-Beliefs

A dictionary definition of self-belief is “the belief that you can do things well” (Macmillan Dictionary). Self-beliefs are part of a broader category of self-assessments in psychology. In our work to date, four psychological constructs that can be classified as self-beliefs have shown noteworthy correlations with academic achievement. They follow the “*predictability gradient*” hypothesis—i.e., they can be ordered in terms of the size of their correlations with other measures (see Stankov 2013; Stankov and Lee 2017):

*Self-concept* refers to the beliefs we hold about our competencies and attributes (Marsh 2007). In PISA studies, a specific mathematics self-concept was measured with statements like, “I am good at math”, and “I learn mathematics quickly”. This construct tends to have a borderline correlation with achievement, but in Stankov and Lee (2017) Self-concept’s correlation with mathematics achievement score was medium at r = 0.26 (or between r = 0.20 and r = 0.30).

*Test anxiety* is a worry, dread, and fear of failure in test situations. According to the meta-analysis reported by von der Embse et al. (2018) this construct has a negative correlation, between r = −0.13 and r = −0.40, with academic achievement. In our work, test anxiety had an r = −0.36 with achievement scores in mathematics (Lee and Stankov 2018).

*Self-efficacy* is a ‘can do’ self-belief (Bandura 1986), typically measured with items that ask whether a person thinks that they can do a particular task, such as calculating the square footage of a room or solving an equation like 3x + 5 = 17. Schneider and Preckel (2017) point out a large effect size of self-efficacy for predicting achievement. In our own work, its correlation with mathematics achievement scores is large and ranges between r = 0.30 and r = 0.45 (Lee and Stankov 2018).

*Confidence* (labeled self-confidence in our earlier work) was inspired by the work of Kahneman (see Kahneman et al. 1982). It is assessed by asking a person to state how confident they are that the answer to a just-solved test item was correct. After each item in a cognitive test, the participant indicates confidence on a percentage scale from 0 to 100%—i.e., “I am ___% confident that my answer is correct”. This measure was not used in PISA or TIMSS surveys but was a part of the cognitive psychology’s confidence research over the past 30 years. Correlations between confidence and accuracy scores on cognitive tests are very large and range between r = 0.45 and r = 0.65 (Lee and Stankov 2018). Clearly, among the four self-beliefs, confidence is the best predictor of cognitive performance.

It can be noted that the *predictability gradient* hypothesis (Stankov 2013; Stankov and Lee 2017) corresponds to the specificity of the questions that capture the constructs: a. Self-concept refers to a general belief about oneself and it has the lowest correlation with achievement; b. Questions about self-efficacy refer to a belief that one can solve a particular task within a specific subject matter (e.g., mathematics) and has an in-between correlation with achievement; and c. Confidence refers to a particular just-completed test item and has the highest correlation with achievement. Also, the mathematics self-concept is not expected to correlate with the language self-concept—i.e., saying that one is good at mathematics does not imply that they are good at language use. Self-efficacy also tends to be specific—i.e., saying that one can solve an equation does not imply that the person can write a good essay. As we shall see shortly, confidence in having solved an item from one domain implies that the person is likely to express similar levels of confidence about being able to solve an item from another domain as well.

## 4. Special Role of Confidence in Cognitive Processing

The study of individual differences in confidence has produced two findings of importance to education. Stankov et al. (2014) reported that confidence measures based on different cognitive tests tend to define a strong common factor in much the same way that intelligence tests define a general factor ‘g’ of intelligence. This means that confidence obtained with, say, a test of mathematics can predict confidence in some other area, such as a test in science. They also point out that confidence has a small incremental predictive validity for achievement over and above corresponding cognitive measures themselves. Therefore, the assumption is that confidence will act in the same way in real-life decision-making.

The study of confidence is closely linked to three areas of cognitive and educational psychology. First, confidence is seen as an important ingredient of the *decision-making process*, particularly under conditions of uncertainty (see Jackson et al. 2017). We all need to be well-calibrated—i.e., show realism in the assessment of our performance in school and in life. Otherwise, psychological reactions to maladaptation may affect our behavior and well-being. Poor calibration is commonly manifested as overconfidence bias, which is arguably the most important bias in human reasoning (see Teovanović et al. 2015; Moore and Healy 2008). This bias represents the difference between the average confidence rating over all items in the test and the percentage of correctly solved items. It is typically found that people think that they have solved more items than they did, hence the label ‘overconfidence’ bias. In mathematics, for example, Morony et al. (2013) reported that males have a higher overconfidence bias than females in eleven European and Confucian countries.

Second, post-item measurement of confidence is seen as an aspect of the *monitoring* component of *metacognition* (see Fleming 2024). In other words, reflecting on the accuracy of each of the just-provided answers informs a test taker about their level of performance over a series of items. This information can then be used to modify test-taking strategies. Recently, it has been shown that metacognitive monitoring is a strong predictor of the giving-up behavior in test-taking (Law et al. 2022) and may play a role in competing (or dual) task performance. There is also evidence that confidence itself may play an important role in collective decision-making (Blanchard et al., forthcoming).

Third, in educational psychology, the term “confidence” appears in relationship to what we refer to as “self-efficacy” in the present review. In the theory of self-regulated learning (Zimmerman 2002), self-efficacy plays a central role in setting goals, planning strategies, monitoring progress, and managing attention and motivation. It reflects self-observed performances against some standard, such as one’s prior performance or an absolute standard of performance. One’s self-efficacy is updated through self-judgment where learners may build or undermine confidence for future tasks. An abundance of research (Jackson et al. 2017; Lee and Stankov 2016; Morony et al. 2013; Stankov et al. 2014) suggests that this kind of self-evaluation may be a particularly good predictor of life outcomes, including academic achievement.

## 5. Test Anxiety and ‘Dark’ Personality Traits

The presence of test anxiety in the list of noteworthy self-beliefs that have negative correlations with achievement suggests that psychological measures of aberrant rather than typical behavior captured by the Big Five may need to be considered. Indeed, Borghans et al. (2016) reported the results based on measures of self-reports and teachers’ assessment of disorderly activity, antisocial behavior, grit, self-esteem, locus of control, and neuroticism in support of the claim that personality may be more important than cognitive ability for the prediction of life outcomes, including academic achievement. Apart from the role of Neuroticism, these life outcomes are not captured well by the Big Five scales. This implies that what are sometimes called ‘Dark’ personality traits (see Paulhus and Williams 2002) which act as impediments to cognitive functioning need to be considered more seriously.

Originally, the ‘Dark’ label was attached to three traits—narcissism, Machiavellianism, and psychopathy (lack of empathy and remorse)—which share some common malevolent features. Nowadays, ‘Dark’ occasionally also includes dispositions that resemble mild clinical symptoms. For example, what Eysenck (1992) referred to as the personality trait of Psychoticism, which is characterized by aggressiveness and interpersonal hostility and linked to schizophrenia. A more recent development along similar lines was a measure of proneness to psychotic-like experiences and behaviors conceptualized as Disintegration. A sample item reads, “Sometimes I feel like someone else inside of me makes decisions instead of me.” (Knežević et al. 2017). The Disintegration measures may produce higher (negative) correlations with life outcomes and intelligence than the Big Five scales of personality. It is also important to note that both Psychoticism and Disintegration measures are applicable to non-clinical populations and can be administered together with measures of intelligence and personality.

## 6. Potential Research and Practical Implications

Knowledge about predictive validity of non-cognitive constructs can be used to further research aiming to develop educational enhancement procedures. Our review suggests that many non-cognitive predictor variables that have been extensively featured in the literature are not performing a sufficiently good job of predicting academic achievement even though their correlation coefficients may indicate statistical significance. A caveat should be noted. The low correlation between a particular non-cognitive variable and academic achievement does not imply that the same variable will be a poor predictor of other life outcomes, such as well-being, health, and business accomplishments. Below, we highlight research and practical implications for the variables reviewed in this paper.

*Low predictability variables*: An effective approach for the variables with *low* correlations may be to discard them or reduce their use. Steps towards removing redundant and inefficient predictors appear to have started in PISA. After 20 years of use, items measuring self-concept were removed from the PISA 2022 surveys, reportedly due to their conceptual and empirical overlap with self-efficacy, and the fact that self-concept showed a comparatively smaller correlation with achievement than self-efficacy. Furthermore, reducing the role of the Big Five personality scales and some measures based on the Big Five model (i.e., Social and Emotional Skills) may be an appropriate direction when it comes to the understanding of student achievement. Instead, an expansion of personality models may be considered. The ‘Dark’ personality measures like Psychopathy or Disintegration that may hinder attention and cognitive functioning may be useful replacements.

*Moderate predictability variables*: Two variables stand out in this category. Moderate correlations between self-efficacy and performance in specific cognitive domains (e.g., mathematics) suggest that praise and encouragement can be beneficial, as they may enhance motivation to invest effort in these areas. Similarly, during adolescence and the school years, low self-efficacy in certain domains (e.g., the arts) may call for encouragement to persevere.

The other variable showing moderate correlation with achievement was socio-economic status (SES). In PISA, a family’s SES scale is a composite consisting of several scales related to families’ economic, social, and cultural status, based on education (both father and mother), occupation (both father and mother), and home possessions. Its predictability for academic achievement is based on the common variance of several SES-related scales, while its individual components also showed moderate effect sizes for academic achievement. For example, cultural possessions (classic literature, books of poetry, and works of art) as part of family SES showed a moderate effect size, suggesting that such items in the household may be beneficial for students’ achievement (Lee and Stankov 2018). Soon, the availability of digital tools based on artificial intelligence will play a greater role in this category.

Given the example of SES measures, it may be useful to consider some of the variables that have shown low predictability for possible development of a composite variable, which may have improved predictability over its individual components. For example, four scales of the Conscientiousness/Task Performance domain from the OECD (2024) Social and Emotional Skill study—i.e., responsibility, self-control, persistence, and achievement motivation—all have correlations slightly above r = 0.10 with achievement. Research efforts to develop a composite scale from these variables may be a move in a meaningful direction.

*High predictability variables*: High correlation between confidence and achievement is perhaps the most promising source of information for the choice of intervention at present. As mentioned above, two findings are particularly relevant. First, the presence of a broad factor that arises from attaching confidence ratings to tasks that involve different abilities and cognitive processes suggests that assessing confidence in one domain (e.g., mathematics) may transfer to another domain (e.g., arts, language, or physical activity). Therefore, information about confidence ratings and providing feedback about the achieved realism of these ratings in one domain may be used to improve calibration and reduce overconfidence bias in other domains (Stankov et al. 2009). Second, providing confidence ratings obtained through a test is an important aspect of the metacognitive process of monitoring. This is seen as the main source of information for metacognitive control that plays an important role in decisions about the choice of strategies—e.g., deciding whether to give up working on a problem—in decision-making (Law et al. 2022).

## 7. Concluding Comments

This paper reviews empirical findings to date about the role of non-cognitive processes in academic achievement. Many variables used in educational and psychological research have low predictive validity and the list includes the Big Five measures of personality. Economists’ arguments about the relative roles of intelligence and personality may still be premature at this stage (Stankov 2023). At the minimum, there is a need to expand the domain of personality and develop new psychological measures whose impact is like that of self-beliefs. Further attempts to increase the predictability of a range of psychological and non-cognitive variables should continue.

The strongest non-cognitive predictors, thus far, appear to be the self-beliefs variables of self-efficacy and confidence, particularly the latter. Educationists should take steps to develop ways to enhance these self-beliefs skills. Measures of confidence from different cognitive tests define a separate factor akin to the general factor of intelligence. In addition, confidence is an important aspect of metacognitive control processes that play a major role in decision-making. Additional work will be needed to explore the role of confidence in life outcomes other than academic achievement.

Further empirical research to clarify how moderating factors—such as national development status, educational systems, and cultural attitudes— may influence the predictive power of non-cognitive variables will be necessary. Large-scale datasets (PISA, TIMSS) and cross-national survey results suggested that cross-cultural differences in self-beliefs are small, whereas differences related to socio-economic status (SES) are somewhat more pronounced (Stankov and Lee 2016). At the national level, SES exerts differentiated effects on student achievement (Lee and Borgonovi 2022): parental education and occupation are stronger predictors in affluent countries, while material resources (e.g., books at home) are more predictive in less affluent contexts. Moderating factors additional to those related to cross-cultural differences will also need to be examined. For example, negative predictability of the ‘Dark’ personality traits suggests that efforts to increase psychological resilience in the population may be justified.

Overall, the lessons to date indicate that, while non-cognitive predictors of life outcomes cannot be ignored, it is important to recognize the pronounced differences among them in order to better understand and guide life outcomes.

## Data Availability

No new data were created or analyzed in this study.
